# Virophages, polintons, and transpovirons: a complex evolutionary network of diverse selfish genetic elements with different reproduction strategies

**DOI:** 10.1186/1743-422X-10-158

**Published:** 2013-05-23

**Authors:** Natalya Yutin, Didier Raoult, Eugene V Koonin

**Affiliations:** 1 National Center for Biotechnology Information, National Library of Medicine, National Institutes of Health, Bethesda, MD 20894, USA; 2 URMITE, Centre National de la Recherche Scientifique UMR IRD 6236, Faculté de Médecine, Université de la Méditerranée, 27 Boulevard Jean Moulin, Marseille, Cedex 5 13385, France

## Abstract

**Background:**

Recent advances of genomics and metagenomics reveal remarkable diversity of viruses and other selfish genetic elements. In particular, giant viruses have been shown to possess their own mobilomes that include virophages, small viruses that parasitize on giant viruses of the Mimiviridae family, and transpovirons, distinct linear plasmids. One of the virophages known as the Mavirus, a parasite of the giant *Cafeteria roenbergensis* virus, shares several genes with large eukaryotic self-replicating transposon of the Polinton (Maverick) family, and it has been proposed that the polintons evolved from a Mavirus-like ancestor.

**Results:**

We performed a comprehensive phylogenomic analysis of the available genomes of virophages and traced the evolutionary connections between the virophages and other selfish genetic elements. The comparison of the gene composition and genome organization of the virophages reveals 6 conserved, core genes that are organized in partially conserved arrays. Phylogenetic analysis of those core virophage genes, for which a sufficient diversity of homologs outside the virophages was detected, including the maturation protease and the packaging ATPase, supports the monophyly of the virophages. The results of this analysis appear incompatible with the origin of polintons from a Mavirus-like agent but rather suggest that Mavirus evolved through recombination between a polinton and an unknownvirus. Altogether, virophages, polintons, a distinct *Tetrahymena* transposable element Tlr1, transpovirons, adenoviruses, and some bacteriophages form a network of evolutionary relationships that is held together by overlapping sets of shared genes and appears to represent a distinct module in the vast total network of viruses and mobile elements.

**Conclusions:**

The results of the phylogenomic analysis of the virophages and related genetic elements are compatible with the concept of network-like evolution of the virus world and emphasize multiple evolutionary connections between bona fide viruses and other classes of capsid-less mobile elements.

## Background

The rapid advances of genomics and metagenomics lead not only to the rapid growth of sequence databases but to discovery of fundamentally novel types of genetic elements. The discovery and characterization of giant viruses that infect unicellular eukaryotes, in particular members of the family Mimiviridae infecting amoeba, over the last decade revealed a remarkable new class of agents that are typical viruses by structure and reproduction strategy but exceed many parasitic bacteria in size and genomic complexity [1-6]. Much like bacteria, the giant viruses (sometimes called giruses) possess their own parasites and their own mobilomes, i.e. communities of associated mobile genetic elements [7]. The first virus infecting a giant virus, the Sputnik virophage, was isolated from a mimivirus-infected acanthamoeba and shown to replicate within the mimivirus factories and partially inhibit the reproduction of the host mimivirus [8,9]. The second isolated virophage, named Mavirus, is a parasite of the *Cafeteria roenbergensis* virus (CroV), a distant relative of the mimiviruses [10,11]. The third virophage genome was isolated from the Antarctic Organic Lake (hence OLV, Organic Lake Virophage) where it apparently controls the reproduction of its virus host that originally has been classified as a distinct phycodnavirus [12] but according to a more detailed recent phylogenetic study, is actually more closely related to the family Mimiviridae [13]. Very recently, 5 additional genomes of putative virophages have been assembled from metagenomic sequences [14]. Four complete genomes, those of Yellowstone Lake Virophages (YLSV1-4), appeared to be related to OLV, whereas the fifth, nearly complete one, the Ace Lake Mavirus (ALM), appeared to be a relative of the Mavirus [14].

The three well-characterized virophages possess small isocahedral virions and genomes of 20 to 25 kilobase encoding 21 to 26 proteins each. Although the virophages are similar in genome size and structure and are generally construed as related, only a minority of the virophage genes are homologous. The rest of the genes show diverse phylogenetic affinities suggestive of chimeric origins of the virophages [8,11,12].

Analysis of the Mavirus genome [11] resulted in the unexpected discovery that this virophage shared 5 homologous genes with the large, self-replicating eukaryotic transposable elements of the Maverick/Polinton class (hereinafter Polintons). The Polintons that are scattered among genome of diverse eukaryotes and reach high abundance in some protists, such as *Trichomonas vaginalis*, have long been considered ‘virus-like’ transposons because of their large size (20 kb and larger) and the presence of several genes that are common in viruses but not in other transposable elements such as B family DNA polymerase (PolB), packaging ATPase (ATPase) and protease (PRO) [15-18]. The Mavirus shows by far the closest affinity with the Polintons among the currently known viruses, and accordingly, it has been proposed that the Polintons evolved from the virophages [11].

In addition to the virophages, the giant viruses host several other groups of mobile elements. These include self-splicing introns, inteins, putative bacterial-type transposons and the most recently discovered novel linear plasmids named transpovirons [7]. The transpovirons are highly abundant genetic elements associated with several giant viruses of the Mimiviridae family that contain only 6 to 8 genes two of which are homologous to genes of the Sputnik virophage, indicating multiple gene exchanges within the giant virus mobilome.

We sought to decipher the evolutionary relationships between the three known virophages, the Polintons, transpovirons and possibly other genetic elements and viruses. We come up with a complex network of evolutionary relationships that connect many of these diverse elements through overlapping sets of homologous genes.

## Results and discussion

### Origin and evolution of the virophages

**Figure 1:**
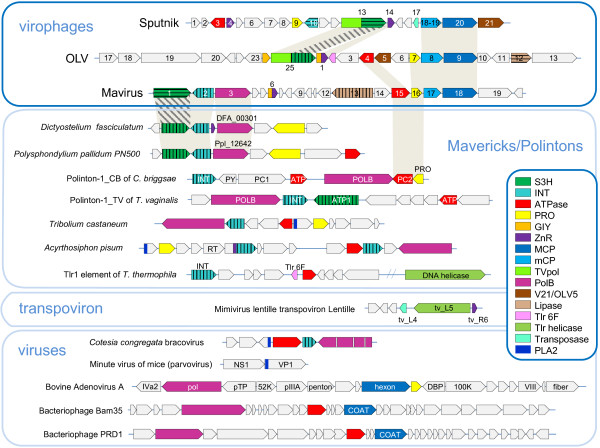
**Comparison of genome architectures of the virophages, polintons, some viruses, and transpovirons.** Homologous genes are marked by same colors. Different hatching patterns are used to mark non-orthologous primase-helicase, integrase, and lipase. Homologous regions are shaded. Reference sequences were extracted from GenBank (*D. fasciculatum,* GI:328871053; *P. palladium*, GI: 281202948; *T. castaneum*, GI:58197573; *A. pisum*, GI:156713484; Mimivirus lentille transpoviron Lentille, GI: 374110342; *C. congregate* bracovirus, GI: 326937614 Minute virus of mice, GI:9626993; Bovine adenovirus A, GI:52801677; Bacteriophage Bam35, GI:38640293; and Bacteriophage PRD1, GI:159192286) and Repbase (Polinton-1_CB and Polinton-1_TV [15,20]). The genome organization of Tlr1was adopted from ref. [18]. *T. castaneum* and *A. pisum* genome fragments are shown as in [21]. PLA2 stands for the phospholipase A2 domain of parvovirus capsid protein. Other color key abbreviations are the same as throughout the text.

To our knowledge, the evolutionary relationships between the virophages so far have not been analyzed in a comprehensive manner. Therefore we performed an exhaustive genomic comparison of the three well-characterized virophages that involved detailed sequence analysis for all predicted virophage proteins (see Methods for details); at this stage, the 5 new virophage genome sequences [14] were not included given potential uncertainties in the genome assembly from metagenomic data. This was followed by phylogenetic analysis of the proteins that showed sufficient evolutionary conservation that, in addition to the three previously characterized virophages, included the 5 new ones. All virophages share 6 homologous proteins or domains: 1) Primase-Superfamily 3 helicase (S3H), 2) packaging ATPase (ATPase), 3) cysteine protease (PRO), 4) Zn-ribbon domain (ZnR), 5) major capsid protein (MCP), 6) minor capsid protein (mCP) (Figure 1 and Table 1). The minor capsid protein initially has not been detected in the Mavirus but direct sequence comparisons supported by gene synteny suggest that MV17 is indeed a highly diverged homolog of the minor capsid protein of the two other virophages (see Additional file 1). The two virion proteins have no detectable homologs outside the virophages (in particular, no environmental homologs; see discussion below) and therefore, per force, are inferred to have evolved from a common ancestor. The recently solved near atomic structure of the Sputnik virion shows that the major capsid protein assumes a diverged double jelly roll structure shared with numerous icosahedral viruses [19].

**Table 1:** Evolutionary provenance of the genes of the three well-characterized virophages

**Gene/protein**	**Domain architecture**	**Predicted activity/function**	**Phylogenetic spread and affinity**	**Representation in environmental sequences**
**Proteins (domains) conserved in all three virophages**				
V9, OLV7, MV16	C5-family cysteine protease	Protease, probably involved in capsid protein maturation	Only distantly related to other proteases from NCLDV, adenoviruses, eukaryotes and some bacteria	No obvious homologs
V3, OLV4, MV15	P-loop ATPase, FtsK-like family	Packaging ATPase	Only distantly related to other ATPases of the FtsK-like family: NCLDV, adenoviruses, diverse phages, bacteria and archaea (DNA pumping during cell division and conjugation)	Abundant moderately conserved homologs
V20, OLV9, MV18	Predicted distorted jelly-roll domain	Major capsid protein	No homologs beyond virophages	None
V18-19, OLV8, MV17	No detectable domains	Minor capsid protein	No homologs beyond virophages	None
V14, V4, MV06 (C-terminal), OLV1 (C-terminal),	C2H2 Zn-ribbon; N-terminal GIY-YIG endonuclease domain in MV06 and OLV1	Unknown	Homologs in transpovirons (closest to V14, Zn-ribbon only), *Phytophtora* and *Dictyostelium* polintons, *P.globosa* virus	Moderately conserved homologs, mostly containing GIY-YIG nuclease domain
V13 (C-terminal), OLV25 (C-terminal), MV01	S3H helicase; N-terminal TVpol in V13 and OLV25	primase-helicase	Sputnik helicase is most similar to bacterial and bacteriophage homologs; the MV01 helicase is most similar to the NCLDV homolog; the OLV helicase is most similar to homologs from bacteriophages and polintons	Numerous conserved homologs including proteins with both TVpol and helicase domains
**Proteins (domains) shared between Sputnik and OLV**				
V13 (N-terminal), OLV25 (N-terminal)	TVpol	Primase and DNA polymerase ([22])	Related to *Micromonas pusilla* and different bacteria	Numerous conserved homologs including proteins with both TVpol and helicase domains
V21, OLV5	No detectable domains	Unknown	No other homologs	None
V6(part), V7(part) OLV13 (part), OLV19 (part), OLV20 (part)	Collagen-like repeats	Adsorbtion on host virus?	V6 is highly similar to mimiviruses, OLV13 - to bacteria; OLV19 has regions similar to OLPV, *T.vaginalis* (phage protein)	Abundant homologs mostly containing collagen domain
**Proteins (domains) shared between OLV and Mavirus**				
OLV1 (N-terminal), OLV24 and MV06 (N- terminal	GIY-YIG endonuclease, fused to C2H2 Zn-ribbon in OLV1 and MV06	Unknown	Close homologs in *Phytophtora* polintons and *P. globosa* virus	Moderately conserved homologs
OLV12 (C- terminal), MV13 (C- terminal)	Lipase 3 domain	Unknown	Homologs in all cellular organisms; Mavirus closest homolog is a *Physcomitrella patens* protein; OLV12 is close to bacterial proteins	Few moderately conserved homologs for each of the proteins
**Proteins (domains) shared between Sputnik and Mavirus**				
V10, MV02	Integrase		Mavirus interase is related to Polintons, Sputnik - to archaeal and bacterial proviruses	Very few homologs
**Sputnik genes with homologs outside virophages**				
V17	Transposase, DNA-binding domain	DNA-binding protein	Closest homologs in transpovirons	Numerous moderately conserved homologs
V16	No detectable domains	Unknown	Homologs in moumouvirus: mv_L1152	none
V12	No detectable domains	Unknown	Highly conserved homologs in Mimiviridae	none
V10	XerD family integrase	Integrase	Closest homologs in archaeal proviruses	Only distant integrases
**OLV genes with homologs outside virophages**				
OLV23	N6 A-specific methylase	DNA methylase	Numerous bacterial homolog	Numerous homologs
OLV16, OLV21	Proline-rich, mucien –like repeats	Unknown (adsorption on virus host?)	Similar repeats in bacteria and eukaryotes	Numerous similar repeats
OLV18, OLV19	Phage Tail Collar Domain	Unknown (adsorption on virus host?)	Closely related to a family of OLPV proteins	Numerous close homologs
OLV2	Uncharacterized domain	Unknown	Homologs in many phycodnaviruses and in Tlr1 element (6Fp)	Abundant homologs with wide range of similarity including very close ones
OLV22	Uncharacterized domain	Unknown	Highly similar to OLPV2, GI:322510937	A few close homologs
OLV12(N-terminal)	Uncharacterized domain fused to Lipase 3	Unknown	Highly similar to Chloroviruses	Numerous close homologs
**Mavirus genes with homologs outside virophages**				
MV20	FNIP repeats	Unknown	Closely related homologs in mimiviruses	Numerous moderately similar homologs
MV04	C2H2 Zn finger	Unknown	No close homologs	None
MV02	RVE family integrase	Integration of Mavirus genome into the virus host genome?	Numerous homologs, closest in Polintons	Numerous moderately similar homologs
MV19, M09	S74 family peptidase (C-terminal), N-terminal glycosylase (?); MV09 has only the N-terminal domain	Unknown	Numerous homologs in phages and bacteria (prophages?); homologs in Marseillevirus, Lausannevirus, *Paramecium* virus, and Polintons (N-terminal only).	Numerous moderately similar homologs
MV13	Lipase (a/b hydrolase superfamily)	Unknown	Homologs in all cellular organisms, closest in plants	Several moderately similar homologs
MV03	B family DNA polymerase	Genome replication	Homologs in all cellular organisms and numerous viruses, the closest homologs in Polintons	No close homologs

The virophage Zn-ribbon is a distinct version of this module that is shared by the virophages and several other groups of mobile elements (see below). In the Sputnik virophage the Zn-ribbon is a stand-alone protein whereas in Mavirus and OLV it is fused to a GIY-YIG endonuclease (GIY), a domain architecture that was detected also in environmental homologs. Conceivably, the ZnR-nuclease fusion is the ancestral version of this protein, with the nuclease lost in the Sputnik lineage. The ZnR domain is too small for reliable phylogenetic analysis (see Additional file 1 for multiple alignments).

**Figure 2:**
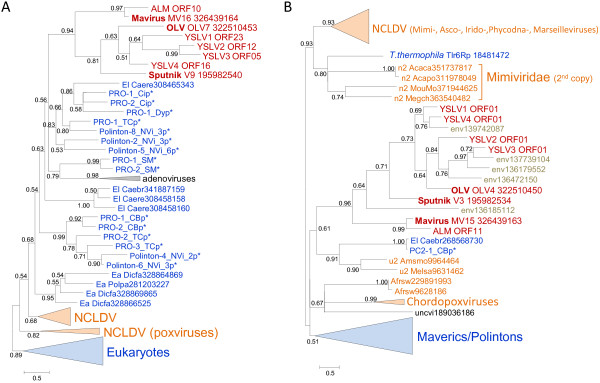
**Phylogenetic trees of conserved virophage proteins. A**, maturation protease**. B**, packaging ATPase. Branches with bootstrap support (expected-likelihood weights) less than 0.5 were collapsed. Sequences marked with an asterisks (*) were taken from Repbase [20]. For other sequences, the species name abbreviation and the GenBank identification numbers are indicated; env stands for “marine metagenome.” Species abbreviations: Acaca, *Acanthamoeba castellanii* mamavirus; Acapo, *Acanthamoeba polyphaga* mimivirus; Afrsw, African swine fever virus; Amsmo, Amsacta moorei entomopoxvirus 'L'; Caebr, *Caenorhabditis brenneri*; Caere, *Caenorhabditis remanei*; Crovi, Crocodilepox virus; Dicfa, *Dictyostelium fasciculatum*; Fowvi, Fowlpox virus isolate HP-438/Munich; Megch, Megavirus chiliensis; Melsa, *Melanoplus sanguinipes* entomopoxvirus; MouMo, Moumouvirus Monve; Orfvi, Orf virus; Popla, *Polysphondylium pallidum* PN500; Tanvi, Tanapox virus; uncvi, uncultured virus; Vacvi, Vaccinia virus Tian Tan. Taxa abbreviations: Ea, Amoebozoa; El, Opisthokonta; n2, mimiviruses; u1, Chordopoxvirinae; u2, Entomopoxvirinae. Color code: Red, virophages; blue, (predicted) polintons and related elements; light brown, NCLDV; gray, unassigned environmental sequences.

The phylogenetic trees for the cysteine protease and the packaging ATPase strongly support the monophyly of the virophages along with the related environmental sequences (Figure 2A,B). In both these trees, Sputnik forms a clade with OLV-YSLV, and the Mavirus-ALM clade is an outgroup to this clade. Taken together, the existence of 5 signature genes including two genes for structural proteins, along with the apparently monophyletic ATPase and the protease required for virion morphogenesis, seems to present sufficient evidence to conclude that despite the diversity of their gene repertoires, the virophages share a common ancestral virus. The existence of such an ancestral virophage is further compatible with the conservation of a three-gene block (cysteine protease and the two virion proteins) between the Mavirus and OLV clades (Figure 1).

**Figure 3:**
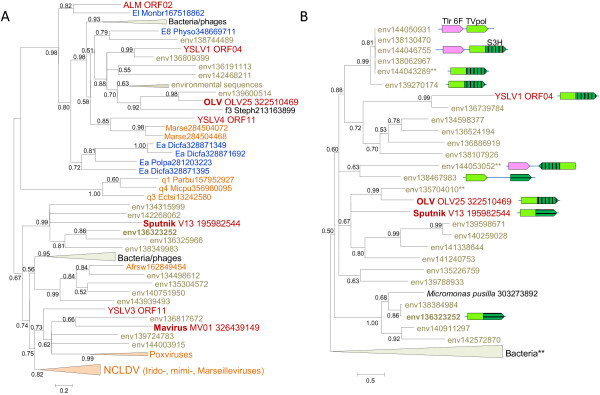
**Phylogenetic trees of virophage S3H helicase: A, helicase domain; B, TVpol domain.** Branches with bootstrap support (expected-likelihood weights) less than 0.5 were collapsed. TVpol domain genome contexts are shown by same color scheme as in Figure 1. TVpol domain reference sequences (marked with two asterisks) were taken from [22]. For other sequences, the species name abbreviation and the GenBank identification numbers are indicated; env stands for “marine metagenome.” Species abbreviations: Afrsw, African swine fever virus; Dicfa, *Dictyostelium fasciculatum*; Ectsi, *Ectocarpus siliculosus* virus 1; Marse, Marseillevirus; Micpu, Micromonas pusilla virus PL1; Monbr, *Monosiga brevicollis* MX1; Mycph, Mycobacterium phage; Parbu, *Paramecium bursaria* Chlorella virus NY2A; Physo, *Phytophthora sojae*; Popla, *Polysphondylium pallidum* PN500; Steph, Stenotrophomonas phage S1. Taxa abbreviations: E8, stramenopiles; Ea, Amoebozoa; El, Opisthokonta; f3, Siphoviridae; q1, Chlorovirus; q3, Phaeovirus; q4, Prasinovirus. The color code is as in Figure 2.

The relationship between the helicase-primase proteins of the virophages is much more complex. All virophages (with the apparent exception of YSLV2 that encodes two predicted helicases of Superfamily 2, ORF5 and ORF10) encode a Superfamily 3 helicase (S3H) domain that in Sputnik, OLV and YSLV1 is fused to the N-terminal domain belonging to a distinct family of polymerases-primases (TVpol) homologous to the bacterial DNA polymerase I [22]. By contrast, in the Mavirus, ALM, YSLV3 and YSLV4, the protein containing the S3H domain encompasses no other recognizable domains. Phylogenetic analysis of the S3H unexpectedly failed to support monophyly of the virophages (Figure 3A; see Additional file 2 for topology testing results). In the tree of the TVpol, the predicted primase domains of Sputnik and OLV virophages belonged to the same clade, albeit with limited support, whereas the putative primase of YSLV1 was lodged in a distinct clade with numerous environmental sequences (Figure 3B). This apparent distinction between the phylogenies of the two domains of the helicase-primase implies a complex evolutionary scenario that might involve multiple origins of S3H domains. Given that the primase-helicase fusion is extremely common among viruses [23,24], the two-domain protein encoded by Sputnik and OLPV could be the form ancestral to virophages whereas the helicase only versions in the Mavirus, ALM, YSLV3 and YSLV4 could have evolved via degradation of the primase domain, perhaps occurring independently in different lineages. This scenario then implies displacement of the helicase domain with homologous domains from different sources (Figure 3A). A recent exhaustive phylogenomic study of the NCLDV has shown that such xenologous gene displacement is common in the evolution of this class of viruses [25].

Sputnik and OLV share two proteins (or domains) that are missing in Mavirus including the primase domain discussed above and an uncharacterized protein V21/OLV5 (Figure 1 and Table 1). In addition, both Sputnik and OLV encode collagen-like repeat-containing proteins that, however, probably were acquired from different sources (Table 1).

**Figure 4:**
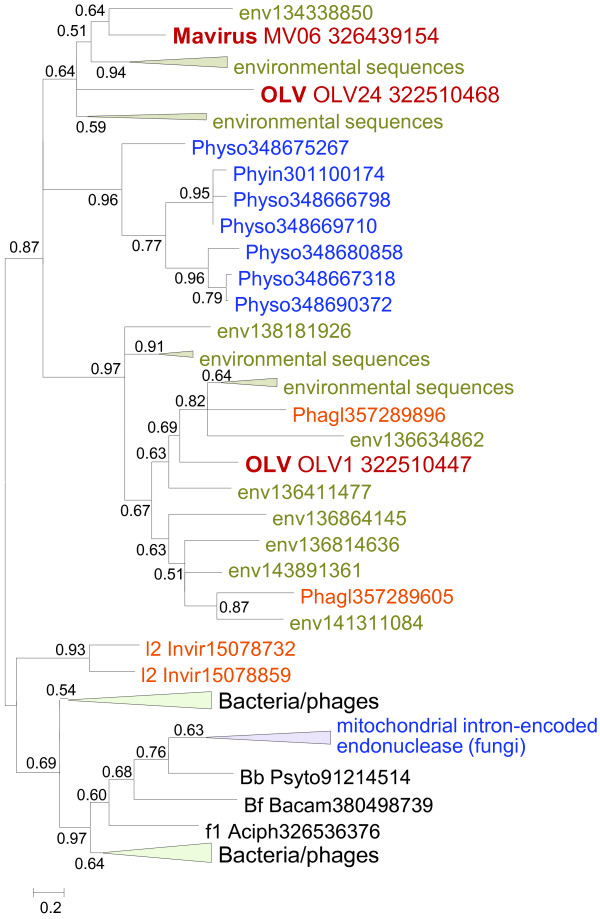
**Phylogenetic tree of the GIY-YIG endonuclease.** Branches with bootstrap support (expected-likelihood weights) less than 0.5 were collapsed. The species name abbreviation and the GenBank identification numbers are indicated; env stands for “marine metagenome.” Species abbreviations: Aciph, Acinetobacter phage 133; Bacam, *Bacillus amyloliquefaciens* subsp. *plantarum* YAU B9601-Y2; Invir, Invertebrate iridescent virus 6; Phagl, *Phaeocystis globosa* virus 12T; Phyin, *Phytophthora infestans* T30-4; Physo, *Phytophthora sojae*; Psyto, *Psychroflexus torquis* ATCC 700755; Taxa abbreviations: Bb, Bacteriodetes/Chlorobi group; Bf, Firmicutes; l2, Iridovirus. The color code is as in Figure 2.

The Mavirus and OLV share two homologous proteins (domains) that are missing in Sputnik. One of these is the GIY-YIG endonuclease domain that is encoded in two genes in OLV and in a single gene in the Mavirus (Figure 1 and Table 1) and is fused to the conserved ZnR that is encoded also in the Sputnik genome, without the endonuclease domain (Figure 1). Phylogenetic analysis of the GIY-YIG endonuclease domain (Figure 4) once again suggests a non-trivial evolutionary scenario. The single endonuclease of the Mavirus belongs to a strongly supported cluster with the OLV homolog that lacks the ZnR (OLV24) whereas the OLV domain fused with ZnR (OLV1) belongs in a well-separated cluster with homologs from some NCLDV and polintons as well as environmental sequences (Figure 4). Thus, the common ancestor of the virophages most likely encoded a GIY-YIG-ZnR fusion. The subsequent evolution in the Sputnik lineage involved loss of the nuclease domain whereas evolution of OLV apparently involved a swap of the two endonuclease domains after acquisition of the second endonuclease gene. The second pair of homologous genes specific to OLV and the Mavirus encodes lipases; apparently, these genes have been acquired by the two virophages independently.

Sputnik and Mavirus exclusively share only one pair of homologous genes that encode a catalytic subunit of integrase with homologs in numerous bacterial and eukaryotic transposons. The Sputnik integrase appears to share a common ancestry with bacteriophage integrases [8], whereas the Mavirus integrase groups with homologs from polintons [11]. Thus, the two virophage integrases, although homologous, are not orthologous and might have been acquired in parallel from elements of different type.

The conservation and the demonstrable monophyly of the two capsid protein genes and the key proteins involved in the virion maturation, the protease and the packaging ATPase, imply that the virophages evolved from a common ancestor that was a bona fide virus. In addition to the genes that are conserved in all virophages, the parsimony principle combined with the phylogenetic tree topologies dictates that those genes that are shared by the Mavirus and either Sputnik or OLV are tentatively assigned to the ancestral virophage as well. In practice, there seems to be only one such gene, the GIY-YIG endonuclease containing a ZnR domain (Figure 1).

**Figure 5:**
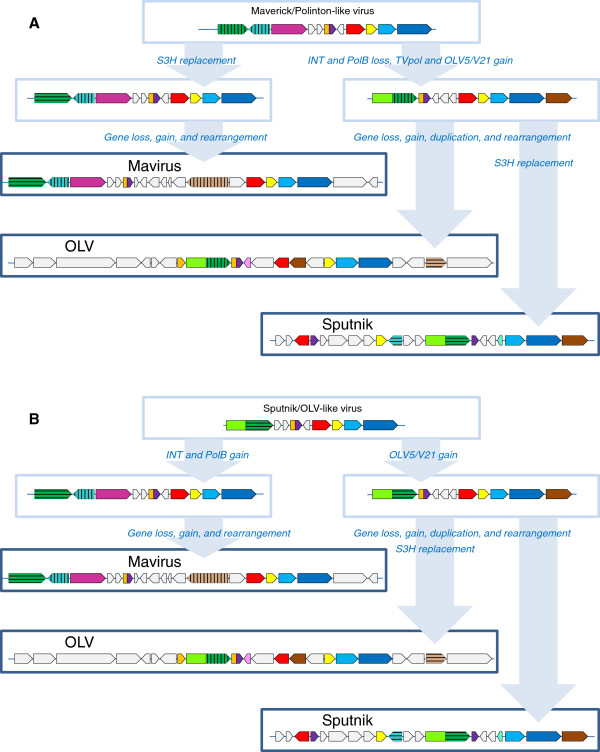
**Two alternative evolutionary scenarios for the virophages. A**, Mavirus/polinton-like ancestor. **B**, Sputnik/OLV-like ancestor. The genes are denoted as in Figure 1.

Beyond the conclusion on the existence of an ancestral virophage, the comparative analysis of the 3 virophage genomes, and in particular the complex history of the helicase-primase gene (see above), seem to be compatible with either of two distinct evolutionary scenarios (Figure 5). Taking into account that in the phylogenetic trees of the conserved virophage genes, the Mavirus consistently forms the outgroup to the Sputnik-OLV clade (Figure 2), the first scenario postulates that the Mavirus resembles the ancestral virophage form (Figure 5A). The ancestral virophage genome would encompass a phage or polinton-like S3H, RVE family integrase (INT), PolB, ZnR, and ATPase, and one or two capsid proteins of probable viral origin. Under this scenario of virophage evolution, the Sputnik-OLV lineage lost the PolB and INT genes and acquired the TVpol domain that became fused to the helicase gene, whereas the Mavirus lineage has undergone replacement of the ancestral helicase gene. After the Sputnik-OLV speciation, the Sputnik helicase domain was replaced as well and a distinct integrase gene was acquired (Figure 5A).

The second evolutionary scenario postulates that the Sputnik-OLV genome architecture including the primase-helicase fusion gene is ancestral to virophages whereas the PolB and INT genes were acquired by the Mavirus lineage along with the loss of the TVpol domain; under this scenario, the displacement of the primase-helicase with a distinct helicase domain occurred in the Mavirus lineage (Figure 5B). This scenario is compatible with the fact that fusion of primase and helicase domain is a common feature of diverse viruses (and related plasmids) of both prokaryotes and eukaryotes that apparently evolved in parallel on multiple occasions [23,24]. A hybrid scenario of virophage evolution whereby the ancestral form possessed both the PolB-INT gene block and the primase-helicase cannot be ruled out either although the combination of a PolB of the protein-primed subfamily with a primase-helicase does not seem to be common.

Regardless of the exact evolutionary scenario, the virophages clearly combine genes from several different sources as noticed in the original report on Sputnik [8] (Table 1). Modularity is a general feature of virus genome evolution [26] but even against this background, the patchiness of the virophages is notable. The contributions of distinct modules with different biological provenances are implied by the fact that closely related environmental homologs (primarily, from marine environments) are readily detectable for some virophage genes, in particular the OLV and Sputnik primase-helicase, but not for those that encode the two virion proteins or the maturation protease (Table 1). As mentioned above, a recent broad survey of metagenomic data from diverse environments yielded homologs of various virophage genes including those for the major and minor capsid proteins that were used as an anchor to assemble the putative new virophage genomes [14], thus revealing limited presence of virophages in specific habitats. It nevertheless seems likely that most of the environmental homologs of the virophage genes do not come from typical virophages but rather from distinct, still poorly characterized mobile elements, (possibly plasmids) that encode primase-helicases homologous to those of Sputnik and OLV [22]. By contrast, the “viral” module of the virophages, with the capsid proteins and the protease, might have come from a group of eukaryotic viruses that is not widely represented in marine environments.

Remarkably, each of the virophages possesses genes that are closely related to homologs from their specific giant virus hosts (Table 1). Moreover, all these apparent host-derived genes encode different repetitive proteins (distinct forms of collagen-like repeats in Sputnik and OLV, and FNIP repeats in the Mavirus) that could be implicated in the interaction of the virophages with their giant virus hosts [9]. The presence of these genes seems to be a striking case of parallelism in virus evolution.

### The evolutionary connections between virophages and polintons

**Figure 6:**
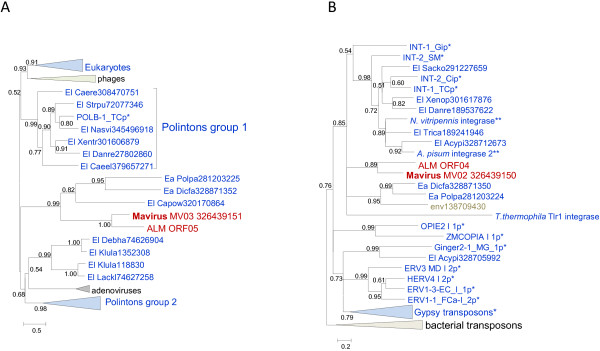
**Phylogenetic trees of Mavirus genes shared with Polintons but not with other virophages. A**, **B** family DNA polymerase. **B**, Catalytic domain of RVE integrase. Branches with the bootstrap support (expected-likelihood weights) less than 0.5 were collapsed. Sequences marked with an astericks (*) were taken from Repbase [20]. For other sequences, the species name abbreviation and the GenBank identification numbers are indicated; env stands for “marine metagenome.” Species abbreviations: Acypi, *Acyrthosiphon pisum*; Caeel, *Caenorhabditis elegans*; Caere, *Caenorhabditis remanei*; Capow, *Capsaspora owczarzaki* ATCC 30864; Danre, *Danio rerio*; Debha, *Debaryomyces hansenii*; Dicfa, *Dictyostelium fasciculatum*; Klula, *Kluyveromyces lactis*; Lackl, *Lachancea kluyveri*; Nasvi, *Nasonia vitripennis*; Polpa, *Polysphondylium pallidum* PN500; Sacko, *Saccoglossus kowalevskii*; Strpu, *Strongylocentrotus purpuratus*; Trica, *Tribolium castaneum*; Xentr, *Xenopus* (*Silurana*) *tropicalis*. Taxa abbreviations: Ea, Amoebozoa; El, Opisthokonta. The color code is as in Figure 2.

The Polintons show notable variability of the gene repertoire but possess a conserved core of 4 genes that consists of PolB, integrase, a C5-family protease and a packaging ATPase (Figure 1). All these core genes have homologs in the Mavirus whereas only the latter two are also found in the Sputnik- OLV branch of virophages. The phylogenetic trees of PolB and INT unequivocally cluster the Mavirus-ALM clade within the Polintons assuming the monophyly of the latter (Figure 6A,B). In the tree of the C5 family proteases, Mavirus forms a strongly supported clade with the other virophages, and this clade again is nested within the polinton-adenovirus clade (the internal branches within this clade are associated with relatively low ELW values but the position of the virophages inside the polintons-adenoviruses is supported by several such branches) (Figure 2A). Finally, the tree of the fourth core gene of the Polintons, the packaging ATPase, includes the virophage clade but fails to retrieve the monophyly of the virophages and the Polintons (Figure 2A). The size of the alignable domain in this case is small, and the reliability of the deep branches in the tree is low. Some Polintons also encode a S3H that falls within the branch of the tree that includes OLV, YSLV1, YSLV4 and ALM along with numerous bacteriophage homologs, but not the Mavirus (Figure 3A). This phylogenetic affinity is compatible with the complex evolutionary scenario for the S3H that became apparent through the comparison of the virophage genomes (see above).

The phylogenetic tree topologies of the virophage genes show much uncertainty, presumably caused by the small size of the conserved domains and their high sequence divergence that probably reflects high and non-uniform evolutionary rates in virophages, other viruses and polintons. Nevertheless, the key observation in the phylogenetic analysis of the genes that are shared by Mavirus with polintons seems to be that Mavirus (or all virophages in cases when they come across as a clade) does not cluster with the polintons as a group but rather falls inside the polinton subtree. This topology of the phylogenetic trees appears incompatible with the origin of the polintons from a Mavirus-like ancestor as previously proposed [11]. Instead, it suggests that either the ancestral virophage evolved via recombination between a polinton and a yet unknown virus (under the scenario in Figure 5A) or perhaps more likely the common ancestor of the Mavirus and ALM evolved via recombination between a polinton and an ancestral virophage (under the scenario in Figure 5B). Of special interest is the strongly supported clade formed by the Mavirus-ALM and a distinct group of polintons from diverse protists in the PolB tree (Figure 6A) that potentially might pinpoint the specific origin of the Mavirus group of virophages.

Under each of the two distinct scenarios shown in Figure 5, the ancestral form is represented as a bona fide virus. The ultimate origin of this virus is not illuminated by the present analysis due to the insufficient resolution of the phylogenetic trees and the extreme divergence of the virophage capsid proteins.

### Bringing in transpovirons and viruses: the virophage-polinton network module

Transpovirons represent a novel class of mobile elements, apparently linear plasmids that so far have been identified only in association with mimiviruses [7]. Remarkably, of the four genes that are shared by different transpovirons, two (ZnR and DNA-binding subunit of transposase) are homologous to genes of Sputnik, the only known virophage parasite of mimiviruses (Figure 1). The ZnR in Sputnik and transpoviron is a stand-alone protein unlike the other two virophages in which it is fused to the GIY-YIG endonuclease (Figure 1). The transposase subunits of Sputnik and the transpovirons form a distinct clade in the phylogenetic tree [7]. These observations imply a direct evolutionary connection between Sputnik-like virophages and the transpovirons, most likely acquisition of the respective genes by the ancestral transpoviron from a virophage.

**Figure 7:**
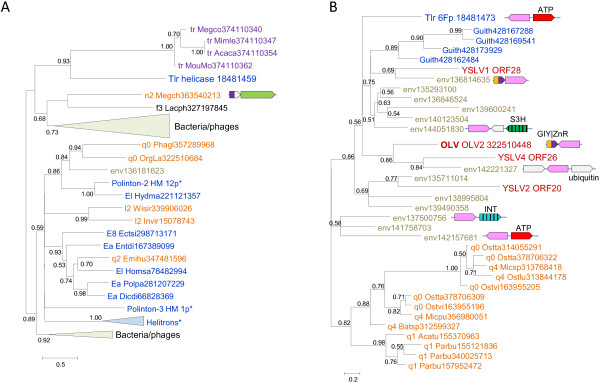
**Phylogenetic trees of Tlr1 proteins shared with transpovirons or virophages. A**, Superfamily 1 helicase. **B**, Tlr1 6F protein. Branches with the bootstrap support (expected-likelihood weights) less than 0.5 were collapsed. Genome contexts are shown by same color scheme as on Figure 1. Sequences marked with an astericks (*) were taken from Repbase [20]. For other sequences, the species name abbreviation and the GenBank identification numbers are indicated; tr stands for transpoviron; env stands for environmental (metagenomic) sequence. Species abbreviations: Acaca, *Acanthamoeba castellanii* mamavirus; Acatu, *Acanthocystis turfacea* Chlorella virus 1; Batsp, Bathycoccus sp. RCC1105 virus BpV2; Dicdi, *Dictyostelium discoideum* AX4; Ectsi, *Ectocarpus siliculosus*; Emihu, *Emiliania huxleyi* virus 84; Entdi, *Entamoeba dispar* SAW760; Guith, *Guillardia theta*; Homsa, *Homo sapiens*; Hydma, *Hydra magnipapillata*; Invir, Invertebrate iridescent virus 6; Lacph, Lactococcus phage 949; Megch, Megavirus chiliensis; Megco, Megavirus courdo7; Micpu, *Micromonas pusilla* virus PL1; Micsp, Micromonas sp. RCC1109 virus MpV1; Mimle, Mimivirus lentille; MouMo, Moumouvirus Monve; OrgLa, Organic Lake phycodnavirus 1; Ostlu, *Ostreococcus lucimarinus* virus OlV1; Ostta, Ostreococcus tauri virus RT-2011; Ostvi, Ostreococcus virus OsV5; Parbu, *Paramecium bursaria* Chlorella virus NY2A; Phagl, *Phaeocystis globosa* virus 12T; Polpa, *Polysphondylium pallidum* PN500; Wisir, Wiseana iridescent virus. Taxa abbreviations: E8, stramenopiles; Ea, Amoebozoa; El, Opisthokonta; f3, Siphoviridae; l2, Iridovirus; n2, mimiviruses; q0, unassigned Phycodnaviridae; q1, Chlorovirus; q2, Coccolithovirus; q4, Prasinovirus. The color code is as in Figure 2.

The Superfamily 1 helicase of the transpovirons has a distinct evolutionary provenance being nested within a branch of the respective phylogenetic tree that includes mostly bacterial and bacteriophage proteins (Figure 7A). Remarkably, however, other than environmental homologs, the closest neighbor of the transpovirons in this tree is the polinton-like transposable element Tlr1 from *T. thermophila*[27], in which the helicase is fused to a distinct GIY-YIG endonuclease. A helicase of the same family is encoded in the unique terminal genomic region of a single mimivirus, *Megavirus chiliensis*[28], in which the adjacent gene encodes a Zn-finger protein homologous to proteins found in some polintons (Figure 7A). In addition to the transpoviron-like helicase, Tlr1 encodes a homolog of OLV2 protein that, upon detailed analysis, was shown to belong to a family of uncharacterized small proteins represented, additionally, in some phycodnaviruses, namely, Chloroviruses and Prasinoviruses as well as the cryptomonad *Guillardia theta* (Figure 7B).

**Figure 8:**
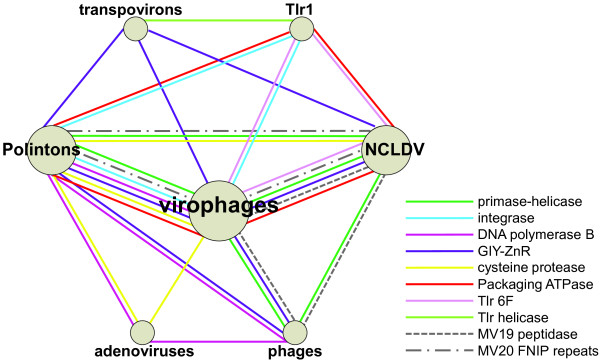
**The virophage-polinton evolutionary network.** Specific groups of bacteriophages that are involved in the network connections: Tectiviridae (PolB); Caudovirales (tailed bacteriophages: S3H and GIY-YIG); cyanophages (MV19 peptidase). Specific groups of NCLDV that are involved in the network connections: Irido-, Mimi-, Pox-, Marseilleviruses (Mavirus S3H helicase); Marseillevirus (OLV S3H helicase and MV19 peptidase); *Phaeocystis globosa* virus and Invertebrate iridescent virus 6 (GIY-YIG); Phycodnaviridae (Tlr 6F); Pox- and Asfarviridae (ATPase), and Mimiviridae (MV20 FNIP repeats).

The evolutionary relationship between virophages, polintons and transpovirons is best represented as a network in which the edges correspond to shared genes (Figure 8). This network also includes at least three distinct groups of viruses, the NCLDV, adenoviruses and an assemblage of bacteriophages. The network is tightly connected, with the edges typically linking the nodes through multiple genes (Figure 8). Clearly, this network is a module of a much large network that connects most of the virus world, primarily through the virus hallmark genes such as S3H, the icosahedral capsid protein or the integrase [26,29]. By its very nature, the network representation of evolutionary relationships lacks directionality. While we concluded that the evolution of the Mavirus branch of virophages involved a major contribution from polintons (see above), it is unclear whether the polintons themselves originated as capsid-less, self-replicating elements or, perhaps more likely on general grounds, evolved from an unknown ancestral virus that lost the capsid.

## Conclusions

The results of the phylogenomic analysis of the virophages, polintons and other related genetic elements reinforce the network character of the evolution of the virus world [26,29]. The distinct groups of elements in this network are connected through different, overlapping sets of shared genes (Figure 8) resulting in a blurred distinction between monophyly and polyphyly. Certain groups, such as the virophages or the NCLDV (recently proposed to be recognized as the order *Megavirales*[30]), can be considered monophyletic in the sense that their common ancestor apparently shared many properties with the current representatives of the respective groups. Nevertheless, even in these groups, subsequent evolution involved acquisition, loss and replacement of a large fraction of genes as demonstrated here for the virophages. Notably, it has been recently shown that the virophages of the *Mimiviridae* have a broad host range and thus can serve as vectors for gene exchanges among the three different groups of mimiviruses [31,32]. The virophage-polinton network (Figure 8) is not isolated from the rest of the virus world but rather is connected to other groups of viruses and virus-like elements through hallmark genes. However, it seems to be a distinct module in the overall network of virus evolution.

Another important outcome of this analysis is the demonstration of multiple connections between bona fide viruses that encode capsid proteins and form infectious viruses and non-viral mobile elements such as transposons. It appears that viruses evolved from non-viral genetic elements and vice versa on more than one occasion even within this relatively small module of the virus evolution networks. These findings imply that capsid-centric concepts of virus evolution [33,34] capture only one, even if important, facet of the virus world history.

## Methods

The protein sequences were extracted from the RefSeq database (NCBI, NIH, Bethesda) [35]. The non-redundant database of protein sequences at the NCBI was searched using the PSI-BLAST program [36]; for proteins of unclear provenance the PSI-BLAST iterations were run until convergence with the E-value cut-off of 0.01 [37]. A separate BLASTP search was run against the environmental protein sequence database (env_nr) at the NCBI. Reference eukaryotic repetitive DNA elements were downloaded from the Repbase database [20], and each virophage protein was searched against the Repbase proteins using BLASTP [36] with the E-value cut-off of 0.1. Nearly identical sequences were eliminated using blastclust (http://www.ncbi.nlm.nih.gov/Web/Newsltr/Spring04/blastlab.html); a representative (the longest) sequence from each cluster was taken. Protein sequences were aligned using MUSCLE [38]; gapped columns (more than 30% of gaps) and columns with low information content were removed from the alignment [39]. A preliminary tree was constructed using the FastTree program with default parameters (JTT evolutionary model, discrete gamma model with 20 rate categories) [40]; the best-fit substitution model was identified using ProtTest [41]; and the final maximum likelihood tree was calculated using TreeFinder [42], with the substitution model found to be the best for a given alignment in the first-round analysis. The following substitution models were identified by ProtTest as the best fit for individual genes for which phylogenetic analysis is reported: protease - WAG + G + F; ATPase - LG + G + F; S3H helicase - Blosum62 + G + F; TVpol - LG + G + F; GIY-YIG endonuclease - RTrev + G + F; PolB - LG + G + F; RVE integrase - Blosum62 + G; transpoviron helicase - LG + G; OLV2/Tlr6F - LG + G.

The branch support values were expressed in Expected-Likelihood Weights (ELW). For S3H helicase, alternative tree topologies were tested with TreeFinder using the approximately unbiased (AU) test [43]. In addition to the TreeFinder, maximum likelihood trees were also computed using the PhyML program [44] with the same alignments and substitution models. The topologies of the PhyML trees were generally compatible with those obtained with TreeFinder but with less resolution and weaker support (see Additional file 3).

## Competing interests

The authors declare that they have no competing interests.

## Authors’ contributions

EVK initiated and designed the study; NY and EVK collected and analyzed data; EVK and DR wrote the manuscript that was read, edited and approved by all authors.

## Supplementary Material

Additional file 1 Multiple alignments of conserved virophage proteins/domains: MCP, mCP, GIY, ZnR, and Mavirus peptidase mv19. Click here for file

Additional file 2 Primase-helicase topology testing. Click here for file

Additional file 3 Multiple sequence alignments and phylogenetic trees computed using TreeFinder and PhyML (Newic format). Click here for file
