# Supplementary material for: Virophages, polintons, and transpovirons: a complex evolutionary network of diverse selfish genetic elements with different reproduction strategies
Source: Virol J. 2013 May 23;10:158. doi: 10.1186/1743-422X-10-158 (PMC3671162; doi:10.1186/1743-422X-10-158)
Supplement: Additional file 1 — Multiple alignments of conserved virophage proteins/domains: MCP, mCP, GIY, ZnR, and Mavirus peptidase mv19. [file 1743-422X-10-158-S1.pdf]

Minor capsid protein (mCP)

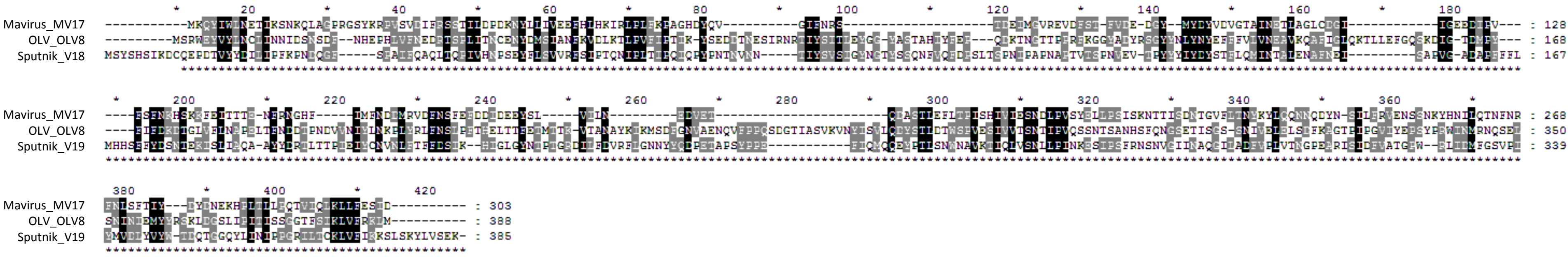

Major capsid protein (MCP)

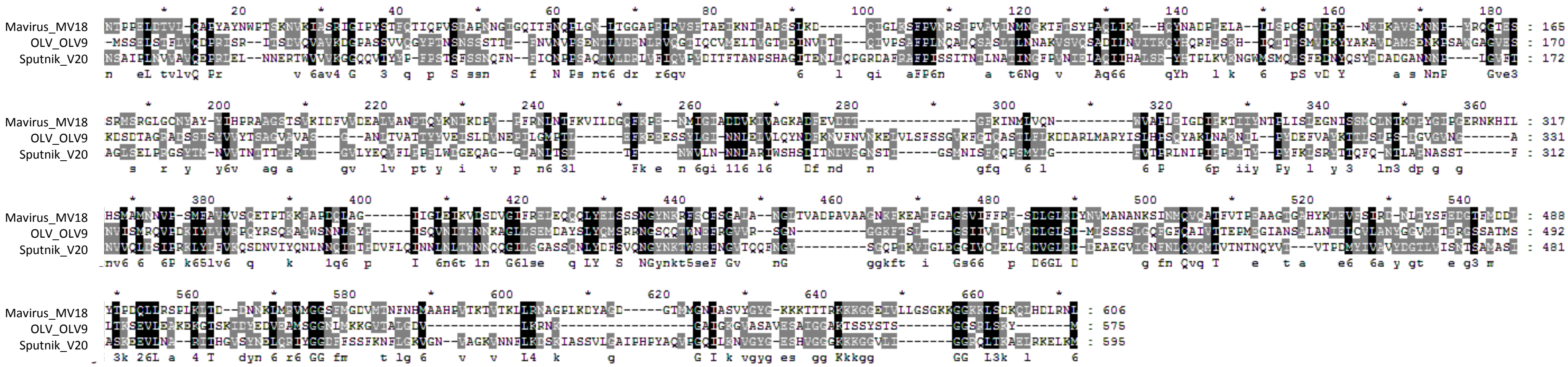

Zn-ribbon domain (ZnR)

```
                                        *         20         *         40         *
env135528457        : SKAKQKYEENKDYLKKKITCE-CGSVIRITDKAKHEKTKKHIAYC-------
env136411477        : ------S---------QLIECQ-CGSKIVKYNYYKHLKTKKHQNYI-------
env136834482        : ----------KEKINEKIDCK-CGKYTHNHQSRHLKSKKHQLYL-------
env136843454        : ------RDEVNKKKREKVICK-CGVSVSKSGLARHRKRGVHQAYL-------
env139737098        : ------RAVNN----FKIECS-CGASVSKPNIARHLKSKKHLDNI-------
env140745199        : ------KEKILEKGKEKVECP-CGSVVRKDNLPKHKKTQKHQNYL-------
env141683978        : ------REKIKEYRSQIIECE-CGSTIQLQEKARHLKSKKHINFT-------
env142032154        : ------RDKILEKKKQKITCE-CGCEVSKAVIYQHKKTKKHQDLM-------
Phyin301100174      : ------RDRLVE----KVICE-CGHYQYRGRSYHFKSQKHIRWM-------
Physo348667318      : ------EDSIKARRRQKYQCE-CSCRYSYGCKTTHLNTKKHQRWL-------
Physo348669710      : ------KERLVA----KITCE-CGHYQYRGRTYHFKSQKHIRWM-------
Physo348690299      : ------KERLVA----KITCE-CGHYQYRGRTYHFKSQKHIRWM-------
Physo348690372      : ------EDSIKARRRQKYQCE-CSGRYSYGCKTAHLKTKKHQRWL-------
Dicfa328871351      : ----MFRKKVLDYKNEKIECE-CGAITSRVNRYRHLKGKIHVNNM-------
Mavirus_MV06        : ------KEKLR----KKNICELCGGSFTHENKSHHLKSKKHQSAL-------
OLV_OLV1_322510447  : ----------KEKIKEKVECKYCAKLLSKQYMSRHHKTCKSKPVK-------
Sputnik_V14         : ------KPKIKKYYSKKIECPICCALYTRSNVTNHKKSQKHIKAINNDLESN
Sputnik_V4          : NGRTYGKKPAKAQPTDRLICEFCGKTYTRSNRSTHRKTEVCKAYQ-------
tv_L3_Megco374110339 : ------KPEDKAKPNDKLICNICGGGYTRANISKHKKTQKHQIFEQMN----
tv_L5_MouMo374110361 : ------NINEKAKPKDKLICDICGGSYTRSNVSKHKNTQKHQIFESMNDKIR
tv_L8_MouMo374110364 : ------KEERKEYYSKKVRCTICGAVYARSNVSNHKKTKKHNDAMDDLEIKY
tv_R5_Megco374110341 : -------------PNDKIKCLICNGSYIRQKKSIHEKTKKHCKAL-------
tv_R6_Mimle374110348 : -------------PSDKIKCLVCNKYYKRNAKSKHEKTKTHQRNL-------
tv_R7_MouMo374110363 : -------------PNDKIICLTCGGSYIRQKNLFMKKLINIQEH--------
zn_Phagl357289605   : EKWHTMKDEKNAERRVKIQCG-CGSQHSFGGTAKHLRTDLHKAWV-------
zn_Phagl357289896   : ------PDKMAVYL-EKIVCD-CGAEVCSKGMKRHLTSIKHMEYL-------
                                                 C  Cg          h     h
```

This Zn-ribbon domain is present in **all three virophages** and **all transpovirons**. In Organic Lake virophage OLV1 and Mavirus protein MV06, ZnF is fused to GIY-YIG endonuclease. Closest full-length homologs are found in *Phytophthora infestans* T30-4, *Phytophtora sojae*, *Phaeocystis globosa* virus 12T, *Phaeocystis globosa* virus 14T, and numerous environmental sequences. Multiple paralogs in Phytophtoras (possible transposable elements).

‘Stand-alone’ ZnF domain is found in Sputnik V14, V4, *Dictyostelium*, and transpovirons.

GIY-YIG endonuclease (GIY) and **Zn-ribbon domain** (ZnR)

Organic Lake virophage OLV1, MV06,
Phaeocystis globosa virus 12T (357289896, 357289605),
Phytophtoras, env seq – full length homologs

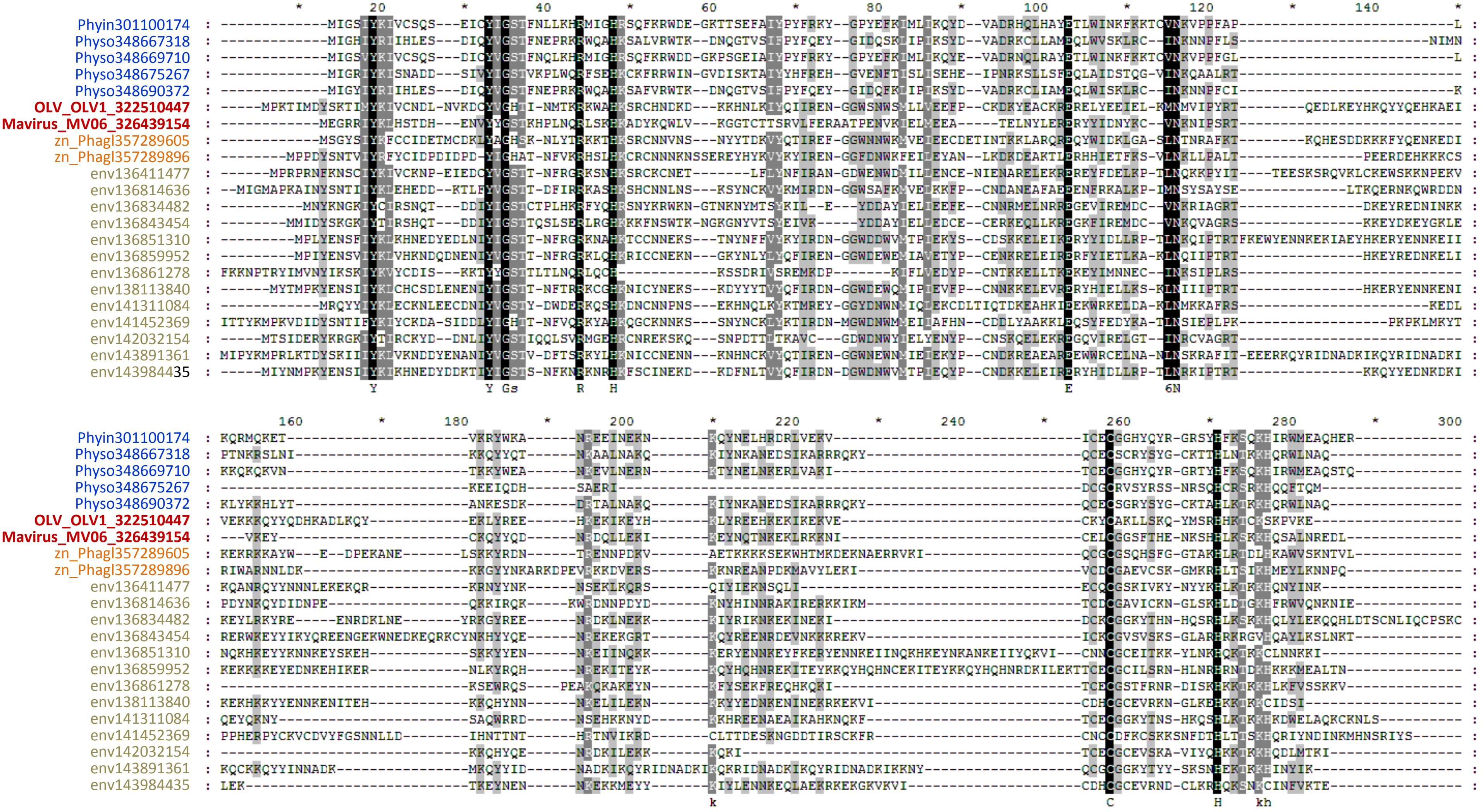

MV6_OLV1_full_len.afa

OLV2/Tlr 6F

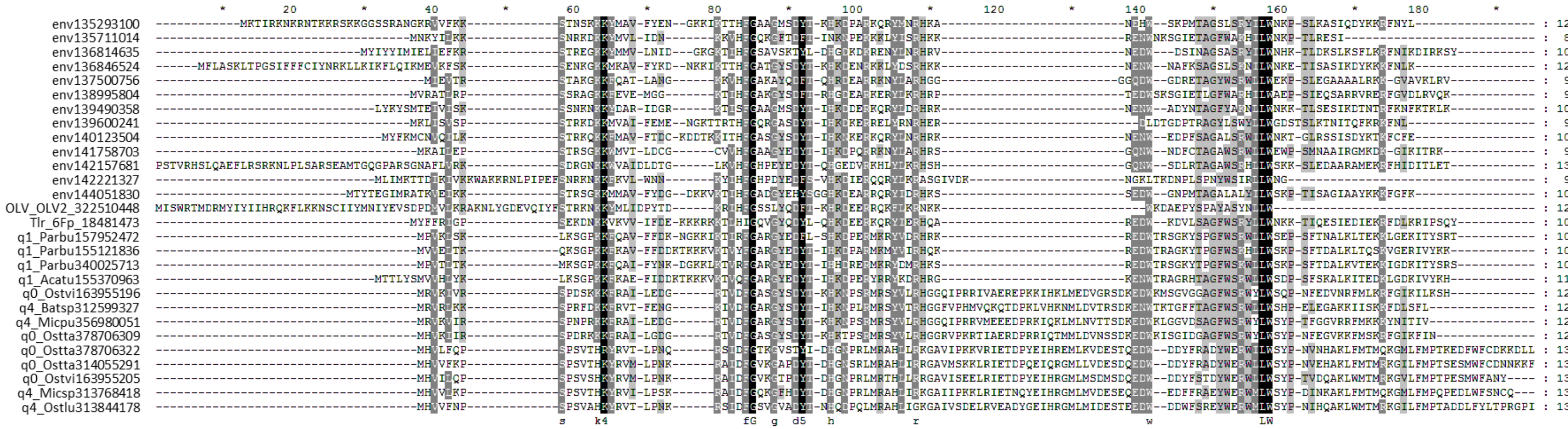
env135293100
env135711014
env136814635
env136846524
env137500756
env138995804
env139490358
env139600241
env140123504
env141758703
env142157681
env142221327
env144051830
OLV_OLV2_322510448
Tlr_6Fp_18481473
q1_Parbu157952472
q1_Parbu155121836
q1_Parbu340025713
q1_Acatu155370963
q0_Ostvi163955196
q4_Batsp312599327
q4_Micpu356980051
q0_Ostta378706309
q0_Ostta378706322
q0_Ostta314055291
q0_Ostvi163955205
q4_Micsp313768418
q4_Ostlu313844178

S74 superfamily Peptidase (Mavirus mv 19, mv9)

Phage (cyanophage?) origin; homologs in Marseillevirus, Lausannevirus, Paramecium virus

```
                      *        20         *        40         *        60         *        80         *       100         *       120         *       140         *       160         *       180
Bb_Dysga33 : ASSPTATNEIAMGASNHSTIRIGNLLSNGSGSWTVTSDRRL-KHDIKPIGQGLDFIKKLKPVEYIYNTGN-----------------GKKSLGFIAQDLQQVMAE---ENMSGYSLVVPT-QGDT----------------------------LGITSTELIPVLTKAIQ------------ :  120
Bb_Flaba16 : VPNASGSHQVRIGNTQISYAGIQ-------VPWTITSDKRW-KDNIRELPYGINMIMQLQPVDYTRKNNE----------------NKTREMGFIAQDLEALLTK---VGYTDQGFLTKD-DDGY----------------------------MSVRYNDIIALLTKAIQ------------ :  114
Bb_Flaba16 : IPDTNNAYRLGNSSSRWIGVWATD------GTINT-SDKRE-KKNIKELNYGLAEVLQMQPVSFNWKNKN----------------NPDLKLGLIAQDLQTLIPEVVKSHTWEKDEISGQLTKKE--------------------------LERLGVYYSDLVPVLINAIKEQQDQIK----- :  128
Bb_Flaba16 : IPDFDDTNVLGNPTQRWRTLYAVN------GTINT-SDRRE-KKNIHDLGYGINEVLQMKPISFNWKNTN----------------NPDLKLGLIAQDLQALIPEVVKSHAWEADEVTGQLTKKE--------------------------LDRLGVYYSDLVPVLIKAIQ------------ :  121
Bb_Lacsp33 : VPFGNGSNQVRVGNTLIAYAGVQ-------VAWDVTSDKRW-KNTIEDSNLGLDFINTLRPVSYFRNNDK----------------TNRIEYGFIAQELKQALQS---SGVKSKSIVSED-SEGM----------------------------LSVRYNDLFSPIVKAIQ------------ :  114
Bb_Leeb186 : GVRIGVSNKIRLGNTAITVVEAQ-------VALSTTSDRRY-KEEIATLPLGLDFINQIRPVEYVRKSNA----------------DNTKEWGVIAQELQQVLAD---TGYDGAGLISEDGSEEH---------------------------YLSVRYTDLIAPMIKAIQ----ELT----- :  119
Bdellovibr : TPDITASKNIGSSTLRWNNIYLS-------NAPDVSSDARL-KKNVKDSDLGLDFVNSLRPVSWTWKDEN---------------QGATEHYGVIAQEAELAIAK---AKGEPSDVIVTH-NEDS--------------------------DSYSVRYTELIAPIIKAVQELYRDLL----- :  124
Bp_Bdeba42 : RPRTDNAFDLGTTTYRWKAVYAVN------GTIQT-SDQRL-KAEVQDLSQGLDFVMALQPKSYKWKSDQ-------------NDPAAKTHWGFMAQELEAQVKR-STASAAPVGLIHHETNSDY----------------------------YGVNYSELIAPVVKALQ------------ :  120
Bp_marga11 : GAVATVSDMVRIGNADVSVFETQ-------LPWTTTSDERL-KESIEPIDGGISFVNDINPVSYQRIGAK----------------EKSTEMGFLAQEVAVVLEA---HGLADSGLVHQS-SPES---------------------------MMTMRYNDLLAPMIKAIQELDAENRQKDAH :  127
MV19_32643 : RPETDNTIDLGYNTFRWKDVYSAN------GTIQT-SDLNK-KKDVNDITKGLDFINTLRPVEYKFKENT----------------SDRVHYGLIAQEVETVFNNLGDSDLTGNATVIKS-QKMIPDPNFIPQSDGTGTIQKTPLIEGEGFDYSLRYTEFIAPLIKAVQ------------ :  144
MV9_10_11  : LEMERLKRDFKALSSKVSNVQPLE------VSGEVKQDLKLNQNNILSIDKGIKFVNELKAVKFK---DD----------------EGNDHYGLVKSEVEKISK----LAGVNVELVKEN-NHLS------------------------------DNLIPLLIKSIQ------------ :  107
Cyanoph372 : IPQGDDTHDLGASNERWDDVYASN------GSINT-SDKNQ-KNTIVESDLGLSFVNKLKPVSYKFNNKT-------------------RTHYGLIAQDVETTLSDISKPTSGFAGFIKED-IPDQ---LYVEDDDIPEGKKVGDVKTAAFTTYGLRYHEFISPLIKAVQ------------ :  139
env1353419 : ----ITNNAFRFYVSGGGQIHATS------TSITAISDQSL-KENIRDIDKGINTILSLQPRRFDWKNGD-----------------GNDIMGFIAQEVEEVMPE------IVHETKYTD-EETK----------------------------LGLKMGDMIPSLVKAIQ------------ :  107
env1359266 : LRLQVQDTVVTIGDVNQPDVDLQVTGD---ITAFKSSDIRL-KDNISPITKALEKVKSISGNTYTKKSD-----------------GSSHTGVIAQEIEALGLP---------GITTTR-RSGY----------------------------LAVDYDKIVPLLIEAIK------------ :  110
env1359698 : ----------------------------------SDRNQ-KNTIIESDLGLDFVNKLKPVSYKWNEDD-----------------GKTHYGLIAQDVEETLLD---IDKTVSDFGAVS-KEDD---------------------------SPMGLSYNEFISPLVKAIQ------------ :   86
env1361103 : ------------------------------IIAFSASDIRL-KSNITSIADAINKVKSLRGVTFEWNENS---------------EHEGPDTGVIAQEVDALELA---------GLVQQR-QNGF----------------------------LAVRYEKLIPVLIEAIK------------ :   86
env1361607 : ------------------------------GFATMPSDERL-KNNIQNIENSISVIEQLRPVSYTWNENM---------------GRKGNDYGLIAQEVEKILPE----LISETDLIFQD-NEEK---------------------------YKTLSYERLVPFLIKSIQ------------ :   92
env1362637 : ASASNQSNQIILGNSSNDDLRCND------TSISGLSDSRD-KTDIVDLPIGLSFVNSIRPVKFKWASRD-------------GNGNDGKIRAGFLAQELQTASAD----ATYLNLVQDD-NPDK----------------------------LCAKYANLIPVMVQAIK------------ :  117
env1377373 : ------------------------------GFATMPSDERL-KNNIQNIENSISVIEQLRPVSYTWNENM---------------GRKGNDYGLIAQEVEKILPE----LISETDLIFQD-NEEK---------------------------YKTLSYERLVPFLIKSIQ------------ :   92
env1387076 : PSSGTATNSVTLGDGNISALRCNQ------TSISSLSDARD-KTDIIDLPVGLDFVNSIRPVKFKWQRRE-------------PDANDGNIRAGFIAQELQSAQID----NEFLDLVHDD-NPDK----------------------------LEAKQANLIPVLVKAIQ------------ :  117
env1400974 : ------------------------------GFATMPSDERL-KHNVQNIENGISIIEQLRPVSYTWNENM---------------GRKGNDFGLIAQEVEKIIPE----LISETDLIFQD-NEQT---------------------------YKTLSYERLVPFLIQSIK------------ :   92
env1402172 : -ITGGIQRVFQVEAAGSGDTYTND------GTISSLSDERV-KTDIISLTDGIDIVQKLRPVTFKYNDTT-----TDEEGRHDFGYKNDKVRYGFIAQEVEKIAPQ---YVETSTRKINNE-EVDD---------------------------FKSLSTTRMIPMLVKAIQ------------ :  127
env1408662 : ------------------------------GTGAVTSDQKL-KENIVDATPKLDELLKVKVRNFNFKEGT----------------GPLTNKRIGFIAQEIETIWPN---MVVNKQDFDEND-ELTG-------------------------TVTKRLKTTDAFVPILVKAIQ------------ :   97
env1412705 : ASSATVSNEITLGNNNVSTLRCNV------QTISSLSDARD-KTDIVDIPTGLDFINDLRPVKFKWQTRD-------------GNGKDGSISAGFLAQDLQKIQKD---SSAEFLNLVMDN-NPDR----------------------------LEAREGQLIPVLVKAIQ------------ :  119
env1416670 : VPSAAATNTVTLGDGNISNLRCAD------TSISSLSDRRD-KKNITNLPEGIDFINKLRPVKFEWATRE------------GLPAKDGTIRAGFIAQELQEAQ-----TGSEHLDLVMDE-NPDK----------------------------LEAKQGKLIPVLVKAVQ------------ :  118
env1421264 : HPSATSSNNVTLGDSNIGALRCNQ------TSITSLSDGRD-KTDVVDLPIGIDFVNSLRPVKFKWQRRE-------------PDANDGKIRSGFIAQELQEVQKD----SDYLDLVMDD-NPDK----------------------------LEAKQGNLIPVMVQAIK------------ :  117
env1429968 : ---ESTDLPLTYNLEVSGSIRTS-------AAVFQSSDERL-KSNILTVTEGLSKIVSSRGVTFD-------------------KDGTNQYGVIAQEIQKVLPE-----------VVSED-NNGY----------------------------LSVNYNGIVGVLIEAIK------------ :  100
env1434515 : PSANNVSNEITLGNSSIASLRCNV------QTISSLSDARD-KTDIVDIPTGLDFINDLRPVKFKWQTRD-------------GNGKDGSISAGFLAQDLQKTQKE---SSAEFLNLVMDN-NPDR----------------------------LEAREGQLIPVLVKAIQ------------ :  119
env1435083 : -----------------------------------SSLSDARD-KTDIVDIPTGLDFLNDLRPVKFKWQTRD-------------GNGKDGSISAGFLAQDLQKTQKD---SSAEFLNLVMDN-NPDR----------------------------LEAREGQLIPVLVKAIQ------------ :   92
Vn_Proph61 : GSVNSNSNVICLGDNSVQNIYCND------TSISS-SDLRD-KADIQNFDHGLAWIKELRPVTYRWDKRSWYTEDPATKGTPDGSKKTNRIHVGFIAQEAIEVEKKFGYGDKKDNMLITNQ-DEDD-------------------------ADPSYGMKYERLIPVLVNAIK------------ :  138
Vx_uncph29 : GSLTTHNNRVCIGDNNISDAYIR-------VDWTVTSDERD-KTDIQDITTGLDFVNQLKPKSFWFKKDR-------------DTNVKHGDKRLGFIAQDILALEGS---D----PVIIDNN-DLDN----------------------------LKYKGEHLVPILVNAIK------------ :  115
                                           sD    k  6      g6  6    p  5                                  G 6aq                                                                        6  a6

                      *        200         *       220         *
Bb_Dysga33 : ----------EQQVTIETQKQTIDKQQQAIDALLRRVEALENKE----------- :  154
Bb_Flaba16 : ----------EQQHIIERQSNDLRTSNKNYEALLTRIEQIEAKISN--------- :  150
Bb_Flaba16 : ----------TLKSTLQNKEKTNQEQSQLLKSLLDRVEALEQSNN---------- :  163
Bb_Flaba16 : -----------------------EQQSIIDSQNNRINNIEAKLNVLIEEKKAS- :  151
Bb_Lacsp33 : ----------QQNDEIETLKAENKTLKLKQQAIENELLEIKKLLLNKK------- :  152
Bb_Leeb186 : ----------EKDKEIQNLKKQVEAQDAKYDALLKRIEALEKAIN---------- :  154
Bdellovibr : ----------DVKAANAAKDLKIREIERKNEEMTKRLEKIEKALNAK-------- :  161
Bp_Bdeba42 : ----------ELYLKVLGVSDRMDQIEKENQNLKNENQEIKAYLCEKDPQARICK :  165
Bp_marga11 : IAAVEQRLSDQQAELLAVVKAQQAQMQAQQSQMEAQSEQIARMEVMLVNDTLVSR :  182
MV19_32643 : -----------------------EITAQNAAIVARVEALENA------------- :  163
MV9_10_11  : -----------------------SISKRLDVIEKGGPSWND-------------- :  125
Cyanoph372 : -----------------------EISTEVETIKTKVAALEAA------------- :  158
env1353419 : -----------------------EIKTELDAAKARIATLESQ------------- :  126
env1359266 : -----------------------EISAKVDTIENQINN----------------- :  125
env1359698 : -----------------------EITAKNDAIEARIKKLEG-------------- :  104
env1361103 : -----------------------EISDKVDSIEQRLNN----------------- :  101
env1361607 : -----------------------EINNKIKEIENK-------------------- :  104
env1362637 : -----------------------EISAKVTAIEAG-------------------- :  129
env1377373 : -----------------------EINNKIKEIENK-------------------- :  104
env1387076 : -----------------------EISVKVAAIEAG-------------------- :  129
env1400974 : -----------------------EINDKIKKIEQK-------------------- :  104
env1402172 : -----------------------EIEARIKTIEG--------------------- :  138
env1408662 : -----------------------EISAKVTAIESAG------------------- :  110
env1412705 : -----------------------EISAKNDAIEARIKTLEES------------- :  138
env1416670 : -----------------------EISAKNDAIAAEVEQLKSQLNN---------- :  140
env1421264 : -----------------------EISAKNDAIVSEIEQLKSQLNN---------- :  139
env1429968 : -----------------------EIKEEVDDIKSKIYTPPN-------------- :  118
env1434515 : -----------------------EISAKNDAIEARIKTLEG-------------- :  137
env1435083 : -----------------------EISEKNDAIEARIKTLEG-------------- :  110
Vn_Proph61 : -----------------------EISSEIDTIKAKIAE----------------- :  153
Vx_uncph29 : -----------------------EISAKVTAIEAG-------------------- :  127
```

Peptidase (Mavirus mv 19, mv9)

With Marseillevirus, Lausannevirus and their best blast hits:

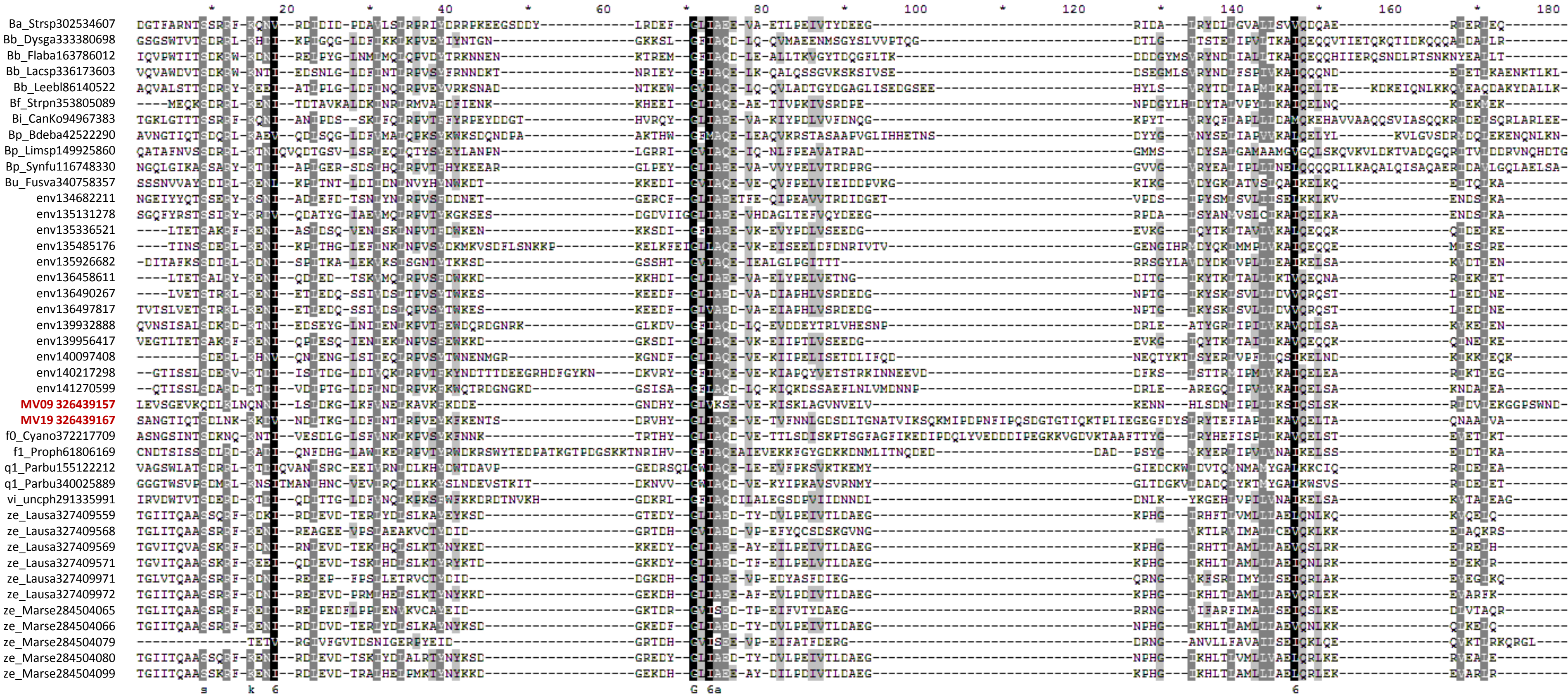

M19 N-terminal

```
                              *        20         *        40         *        60         *        80
MV19_32643 : ---MSISNLLYGNSYKLTCAG-LDAGKNKITNV-APPVDDYDCPNKAYVDSQIDSHSGTLSQVLAAGNDGNGDDMINIGEIQANTLS :  82
Polinton-1 : QTSSSACLKLDGTN---SMSANINLNSHKLINV-VDPTNAQDAATKNYVDRLIPYQHKY--KVLIIAGQSNTYAGREWGLSQSITNV :  81
El_Triad19 : HNEVIKVRGLKGNGYKINDDGDYDLESKRLTNLSSNVVDDSDATNKQYVDSRIDTKVSK--SGDVLYGNLDMNKNCIKNLHDIDPND :  85
El_Trica27 : LSASNAFFKSTPSPFVFTADGDLDLKHHRICNI-NDSTEDKDCANKVYIDLVSKQIYQK---------------------------- :  58
f2_Salph32 : MHVAEEARDLTADNLGVNNDGDLDARGRRIVNV-KDPTLPQDAATKNYVDLVSGGEGSAGKALEELAKPNGAYKIGLGDSTVGDIIG :  86
f2_Vibph32 : LHVAEEARDLAGDSIAVDDNGDLDARGRKIVNL-ADAEDDTDAVNYGMLKKFDNSTFNNAQKAEASANAAKSSENKAKDSELRSIEA :  86
f2_Yerph19 : IHVAEEARDLTADTIGVNNDGNLDARGRKIVNL-AFATSDYDAVPLKQITDRESSVWNAVTKASEQADRSNKEANRSRDEADRAKRE :  86
f2_Salph18 : LHVAEEARDLTADTIGVNNDGNLDARGRRIVNV-ADGVTDGDAISVGQVKRMNQNSWQARNESLQFRNEAEGFRNQASTSKQAAATS :  86
f2_Entph21 : MHVAEEARNLTTDTIGVNNDGHLDARGRRIVNL-ANAVDDRDAVSFGQLKTMNQNSWQARNETLQFRNEAETFRNQAEGFKDESSIN :  86
                              1          g 1    46 N6     d Da    6
```

With more polyntons:

```
                              *        20         *        40         *        60         *        80
Polinton-3 : IHDFNQ-----------ILNDKKLDTIGNDVSEIKKSIIELYDKIKV--IEFFINENSSTTTTTTRAH--------------------- :  55
Polinton-7 : IEGSQQVSRGPPGIGFNFTSDGDFDLEXKRLCNIGEP-RXPNDAITLHSLKVILQTEXNYIASKLXGIGEVIEEYKQQVEKHQLEV--- :  85
Polinton-6 : IKKNEG-DRGPPGFGFKITKDGQYDLDNKRLCNVAAP-HKLNEAVDLGTLHTIHQMEHKKVRDVTDKLREELKDLDSLIEAHRDEM--- :  84
Polinton-4 : SNRSKG-RRGPPGVGYKITADGHFDIGSRRLCNVAEP-LELNDAVNLAVLQRSINSEIRSVHEITSDLRQSIDAVELAVQIAKDEN--- :  84
Polinton-1 : QTSSSA-CLKLDGTN---SMSANINLNSHKLINVVDP-TNAQDAATKNYVDRLIPYQHKYKVLIIAGQSNTYAGREWGLSQSITNV--- :  81
El_Triad19 : HNEVIK-VRGLKGNGYKINDDGDYDLESKRLTNLSSNVVDDSDATNKQYVDSRIDTKVSKSGDVLYGNLDMNKNCIKNLHDIDPND--- :  85
MV19_32643 : MSISNL----LYGNSYKLTCAG-LDAGKNKITNVAPP-VDDYDCPNKAYVDSQIDSHSGTLSQVLAAGNDGNGDDMINIGEIQANTLS- :  82
El_Trica27 : LSASNA-FFKSTPSPFVFTADGDLDLKHHRICNINDS-TEDKDCANKVYIDLVSKQIYQK------------------------------ :  58
f2_Salph32 : MHVAEE-ARDLTADNLGVNNDGDLDARGRRIVNVKDP-TLPQDAATKNYVDLVSGGE-GSAGKALEELAKPNGAYKIGLGDSTVGDIIG :  86
f2_Vibph32 : LHVAEE-ARDLAGDSIAVDDNGDLDARGRKIVNLADA-EDDTDAVNYGMLKKFDNSTFNNAQKAEASANAAKSSENKAKDSELRSIEA- :  86
f2_Salph18 : LHVAEE-ARDLTADTIGVNNDGNLDARGRRIVNVADG-VTDGDAISVGQVKRMNQNSWQARNESLQFRNEAEGFRNQASTSKQAAATS- :  86
f2_Entph21 : MHVAEE-ARNLTTDTIGVNNDGHLDARGRRIVNLANA-VDDRDAVSFGQLKTMNQNSWQARNETLQFRNEAETFRNQAEGFKDESSIN- :  86
f2_Yerph19 : IHVAEE-ARDLTADTIGVNNDGNLDARGRKIVNLAFA-TSDYDAVPLKQITDRESSVWNAVTKASEQADRSNKEANRSRDEADRAKRE- :  86
                                         g 1      6 n6      da    6
```
