# Supplementary material for: Virophages, polintons, and transpovirons: a complex evolutionary network of diverse selfish genetic elements with different reproduction strategies
Source: Virol J. 2013 May 23;10:158. doi: 10.1186/1743-422X-10-158 (PMC3671162; doi:10.1186/1743-422X-10-158)

Primase-helicase (S3H) topology testing

tree	elw	au	Likelihood	tree	constraint description	elw	au	Likelihood
freetree	1.000	0.870	-66820.76	constraint 0	all three virophages with related sequences are monophyletic	0.000	0.000	-66909.7
freetree	1.000	0.852	-66820.76	constraint 1	all three virophages with related sequences are monophyletic	0.000	0.000	-66904.75
freetree	0.991	0.994	-66820.76	constraint 2	OLV and Sputnik with their related env sequences are monophyletic	0.009	0.006	-66885.34
freetree	0.995	0.995	-66820.76	constraint 3	OLV and Mavirus with their related env sequences are monophyletic	0.005	0.000	-66891.99
freetree	0.760	0.756	-66820.76	constraint 4	Sputnik and Mavirus with their related env sequences are monophyletic	0.240	0.244	-66832.73

Only the monophyly of Mavirus and Sputnik was not rejected (AU > 0.05)

Constraint 0

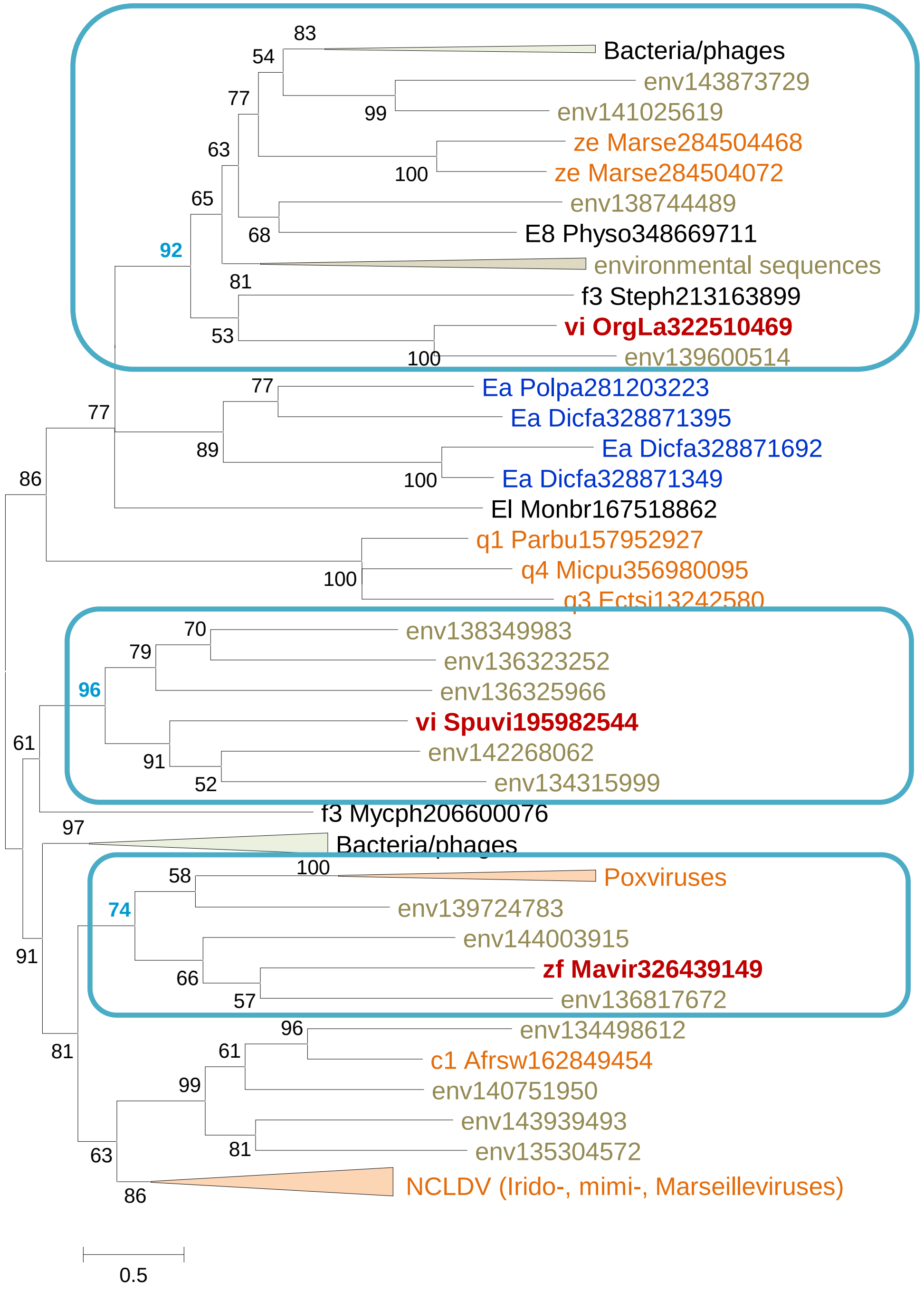

Constraint 1

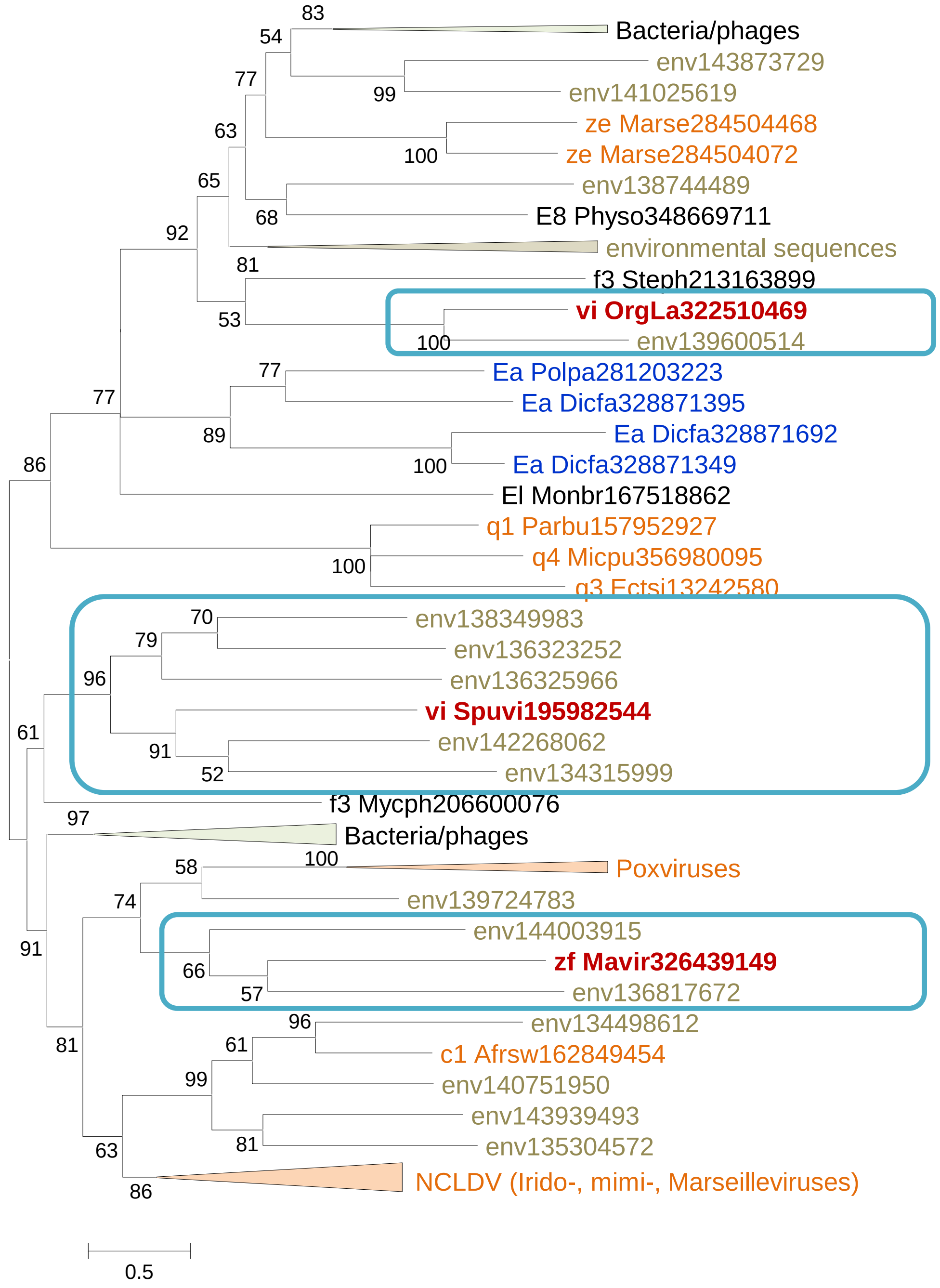

Constraint 2

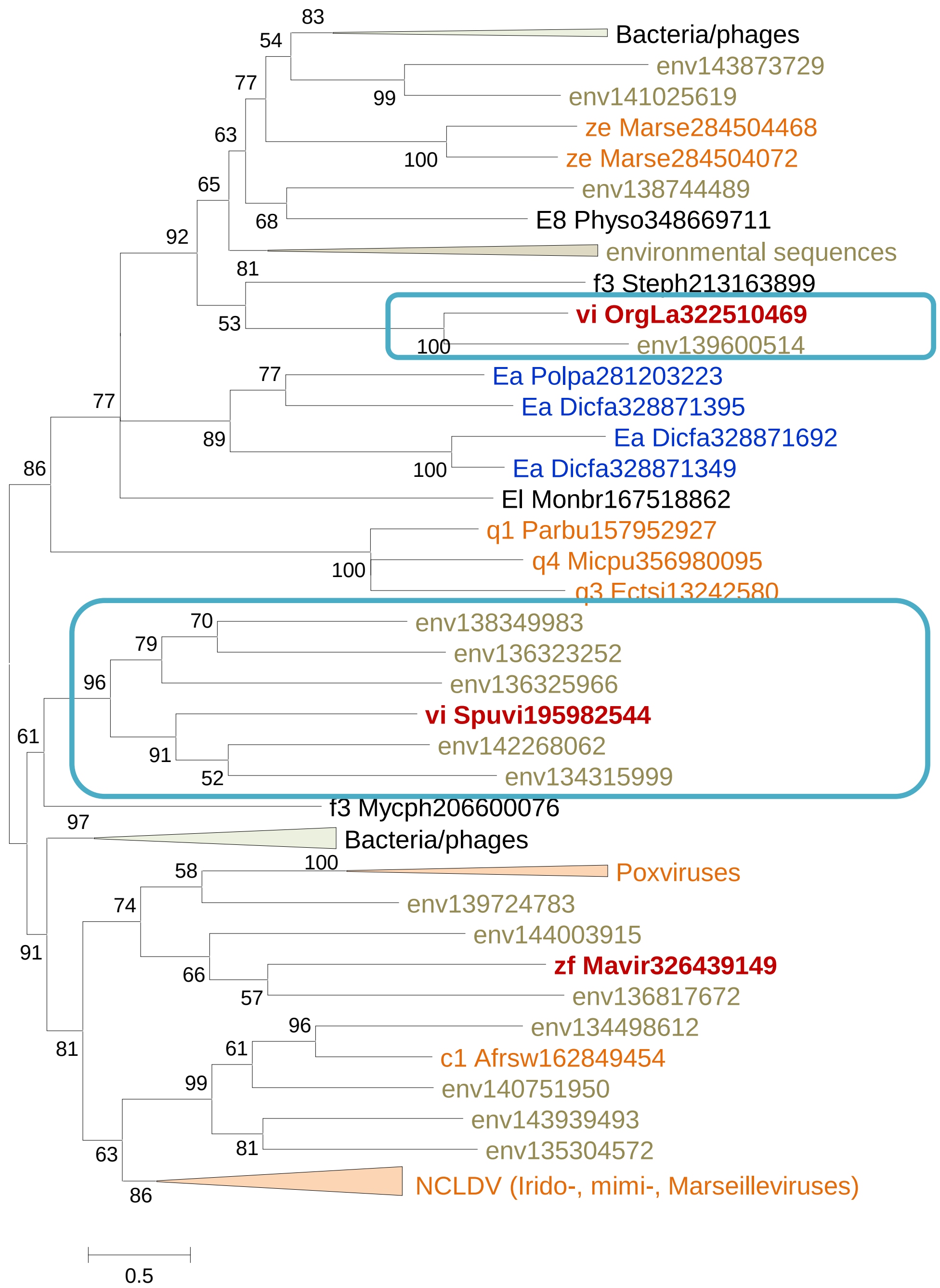

Constraint 3

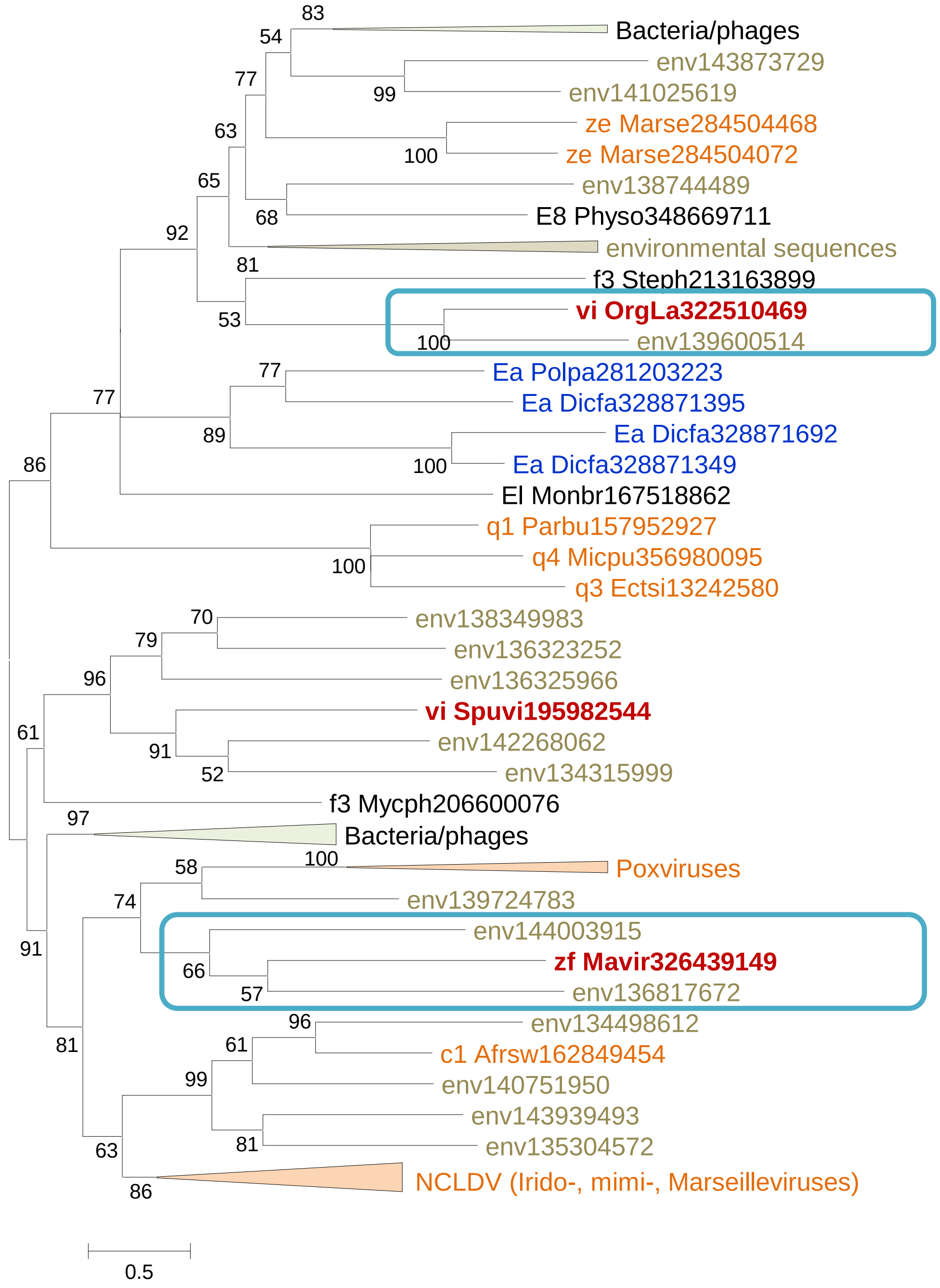

Constraint 4

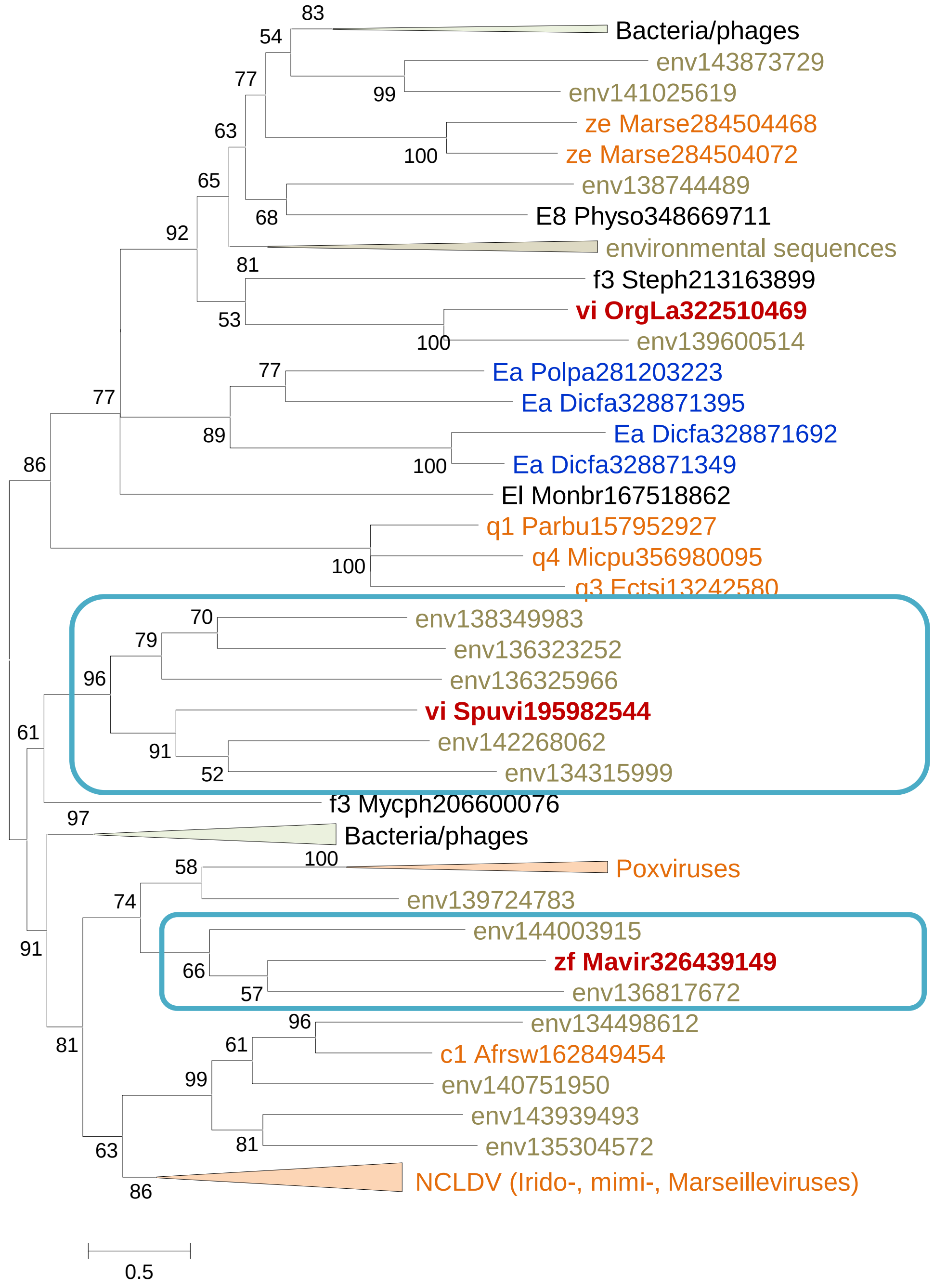

Supplement: Additional file 2 — Primase-helicase topology testing. [file 1743-422X-10-158-S2.pdf]
